# Which appendicitis scoring system is most suitable for pregnant patients? A comparison of nine different systems

**DOI:** 10.1186/s13017-020-00310-7

**Published:** 2020-05-18

**Authors:** Baris Mantoglu, Emre Gonullu, Yesim Akdeniz, Merve Yigit, Necattin Firat, Emrah Akin, Fatih Altintoprak, Unal Erkorkmaz

**Affiliations:** 1 Department of General Surgery, Sakarya University Educating and Research Hospital, Sakarya, Turkey; 2 Faculty of Medicine, Department of General Surgery, Sakarya University, Sakarya, Turkey; 3 Faculty of Medicine, Department of Biostatistics, Sakarya University, Sakarya, Turkey

**Keywords:** Appendicitis, Pregnancy, Score, Predictive value

## Abstract

**Background:**

Acute appendicitis is the most common non-gynecological emergency during pregnancy. The diagnosis of appendicitis during pregnancy is challenging due to changes in both physiological and laboratory variables. Guidelines suggest patients with suspected acute appendicitis should be stratified based on clinical scoring systems, to optimize the use of diagnostic imaging and prevent unnecessary surgery. Surgeons require additional information beyond that provided by imaging studies before deciding upon exploratory laparoscopy in patients with a high suspicion of appendicitis. Various scoring methods have been evaluated for the diagnosis of acute appendicitis. However, there is no consensus on a method to use during pregnancy, and a detailed comparison of existing scoring methods for this purpose has not yet been conducted. The purpose of this study was to evaluate the efficacy of the most popular scoring systems applied to diagnose acute appendicitis during pregnancy.

**Methods:**

This single-center retrospective study included 79 pregnant patients who were admitted to the emergency department with abdominal pain between May 2014 and May 2019. The patients were diagnosed with acute appendicitis and underwent an appendectomy. As a control group, the study also included 79 non-pregnant patients who underwent appendectomy within the last 1.5 years. To ensure that the groups were similar, women in the case group were stratified according to age, and the proportions of women in the strata were determined. The women in the control group were similarly stratified. Women were randomly selected from the strata to prevent bias.

Both laboratory and examination findings required for each scoring method were obtained and assessed separately for each patient. Negative appendectomy rates were evaluated according to pathology results. Categorical variables were compared using the chi-square test. A *p* value < 0.05 was considered to indicate significance. Receiver operator characteristic curve analysis was used to identify the best threshold value and to assess the performance of the test scores in terms of diagnosing appendicitis.

**Results:**

Among all scoring systems, the Tzanakis score was most efficacious at predicting appendicitis in non-pregnant women. The positive predictive value (PPV) of the Tzanakis score was 90.6%, whereas the negative predictive value (NPV) was 46.7%. The RIPASA score performed the best among the scoring systems in pregnant women. It was associated with a PPV of 94.40%, NPV of 44%, and sensitivity and specificity of 78.46% and 78.57%, respectively.

**Conclusion:**

Although the RIPASA score can be used to efficaciously diagnose acute appendicitis in pregnant women, a specific scoring system is needed for diagnosis during the gestation period.

## Background

Acute appendicitis is the most common cause of non-obstetric emergency surgery in pregnant women. Appendicitis occurs in one out of 1500 pregnant women [1]. Furthermore, negative appendectomy rates in females of reproductive age are reported to be up to 26% [2]. The differential diagnosis of acute abdominal pain during pregnancy is more complicated than in typical patients. In addition to symptoms such as nausea, vomiting, and abdominal pain, which are common during pregnancy, an increased white blood cell (WBC) count and limited radiological methods complicate the diagnosis of acute appendicitis [3–5]. Unfortunately, negative appendectomy rates remain relatively high regardless of the testing conducted [6–10].

The main goals in the diagnosis of appendicitis are to reduce negative appendectomy rates, avoid perforation, and protect the patient from unnecessary surgical intervention. A meta-analysis reported that it is essential to make an exact diagnosis and avoid any delay therein [11]. However, another article reported that a delay in the diagnosis of uncomplicated appendicitis of up to 24 h is safe [12]. That article stated that non-operative management through antibiotic treatment is safe in cases of uncomplicated appendicitis. Unfortunately, the rates of abortion and preterm labor are significantly higher in women with appendicitis [13, 14]. Timely diagnosis is critical, as delays may lead to both maternal (1–4%) and fetal (1.5–35%) mortality due to perforation of the appendix [15]. To this end, various scoring methods have been developed based on imaging studies, clinical findings, and laboratory results [16–24]. The present study aimed to evaluate the extent to which these scoring methods are suitable for the diagnosis of appendicitis in pregnancy.

## Methods

This study included 79 pregnant patients who were admitted to the Sakarya University Faculty of Medicine (Serdivan, Turkey) with abdominal pain between May 2014 and May 2019. The patients were diagnosed with acute appendicitis and underwent appendectomy. The study also included a control group of 79 non-pregnant patients who underwent appendectomy within the last 1.5 years. To ensure similar groups, women in the case group were stratified according to age, and the proportions of women in the strata were determined. The women in the control group were similarly stratified. Women were randomly selected from the strata to avoid possible bias. Patients under the age of 20 or older than 45 were excluded from the control group, as well as those with chronic co-morbidities (e.g., hypertension, diabetes mellitus, chronic renal failure, or chronic pulmonary disease). All pregnant patients were examined by the obstetrician both before and after surgery.

Nine appendicitis clinical score methods were used for evaluating the patients. These clinical scoring systems (CSS) included the Alvarado, Eskelinen, Ohmann, AIR, RIPASA, Tzanakis, Lintula, Fenyo-Lindberg, and Karaman systems. Laboratory data such as WBC count, neutrophil count, and C-reactive protein level were collected, as well as examination findings and symptoms. In an Excel file, formulas were prepared separately for each CSS, and the data obtained from each patient were entered into the file so that CSS scores were calculated automatically. A visual analog scale pain score was calculated for all patients hospitalized with a diagnosis of acute appendicitis, who were classified into mild, moderate, and severe pain subgroups according to the literature [25]. This helped with calculating the score for the Lintula system, in which the pain system was graded at three different levels. Negative appendectomy rates were evaluated according to pathology results. The ethics committee of our university approved the study.

### Statistical analysis

Descriptive analyses were performed to provide information on the general characteristics of the study population. The Kolmogorov-Smirnov test was used to evaluate whether the distributions of numeric variables were normal. Accordingly, either independent sample *t* tests or Mann-Whitney *U* tests were used to compare the numeric variables between pregnant and non-pregnant patients. The numeric variables are presented as the mean ± standard deviation or medians with interquartile ranges in square brackets. Categorical variables were compared using chi-square tests. Categorical variables are presented as counts and percentages. A *p* value < 0.05 was considered to indicate significance. Receiver operating characteristic curve analysis was used to identify the optimal threshold values and assess the diagnostic performance of test scores for appendicitis. Analyses were performed using SPSS 22.0 statistical software (IBM Corp., Armonk, NY, USA).

## Results

The median ages in the pregnant and non-pregnant groups were 28 and 26 years, respectively. The mean WBC counts in the pregnant and non-pregnant groups were 14.07 ± 4.5 and 13.43 ± 4.5, and the median C-reactive protein levels were 16.2 [55.03] and 7.64 [41.69] mg/dl, respectively. The left shift in neutrophils was significantly higher in the pregnant group than in the non-pregnant group; 47 (59.5%) vs. 23 (29.1%), respectively (*p* < 0.001). The median total bilirubin level was also significantly higher in the non-pregnant group than in the pregnant group; 0.59 [0.75] vs. 0.47 [0.33] mg/dl, respectively (*p* < 0.001; Table 1).

**Table 1:** Distribution of features related to pregnant and non-pregnant women

Variable	Pregnant (***n*** = 79)	Non-pregnant (***n*** = 79)	***p*** value	Effect size
**Neutrophil**	10.6 [5.6]	10 [5.7]	0.559	− 0.046
**MPV**	7.96 ± 1.49	8.74 ± 1.48	0.001	− 0.524
**Total bilirubin**	0.47 [0.33]	0.59 [0.75]	< 0.001	− 0.324
**Age**	28 [6]	26 [10]	0.236	− 0.094
**CRP**	16.2 [55.03]	7.64 [41.69]	0.28	− 0.175
**WBC**	14.07 ± 4.5	13.43 ± 4.5	0.376	0.141
**PMN ratio**	81.8 [12.2]	78.6 [11.8]	0.078	− 0.140

*MPV* mean platelet volume, *CRP* C-reactive protein, *WBC* white blood count, *PMN* polymorphonuclear leukocyte

Based on the pathology results, 65 pregnant patients (82.3%) had appendicitis and 14 (17.7%) did not. In the non-pregnant group, 66 patients (83.5%) had appendicitis and 13 (16.5%) did not. There was a significant difference between the groups in terms of severity of pain; most individuals in the non-pregnant group (54; 68.4%) reported moderate pain, whereas most in the pregnant group (57; 72.2%) reported a high degree of pain (*p* < 0.001). Findings in both groups were similar in terms of nausea, vomiting, and anorexia. Pregnant patients typically visited the hospital less than 24 h from the onset of symptoms. Based on direct examination of pregnant patients, abdominal guarding and rebound tenderness were significantly more common than in non-pregnant patients (Table 2).

**Table 2:** Distribution of features related to pregnant and non-pregnant women

Variable	Pregnant	Non-pregnant	***p*** value	Effect size*
**Pathology**				
Appendicitis	65 (82.3%)	66 (83.5%)	1	− 0.17
Non-appendicitis	14 (17.7%)	13 (16.5%)		
**USG appendicitis**				
Positive	57 (72.2%)	38 (48.1%)	**0.002**	0.246
Negative	22 (27.8%)	41 (51.9%)		
**Pain severity**				
Mild	1 (1.3%)	17 (21.5%)	**< 0.001**	0.691
Moderate	21 (26.6%)	54 (68.4%)		
High	57 (72.2%)	5 (6.3%)		
Severe	0 (0%)	3 (3.8%)		
**Pain outside the right lower quadrant**				
Positive	5 (6.3%)	11 (13.9%)	0.186	− 0.126
Negative	74 (93.7%)	68 (86.1%)		
**Increased pain in follow-up**				
Positive	25 (31.6%)	35 (44.3%)	0.101	− 0.13
Negative	54 (68.4%)	44 (55.7%)		
**The spread of pain from the umbilicus**				
Positive	26 (32.9%)	48 (60.8%)	**< 0.001**	− 0.279
Negative	53 (67.1%)	31 (39.2%)		
**Vomiting**				
Positive	28 (35.4%)	11 (13.9%)	**0.003**	0.25
Negative	51 (64.6%)	68 (86.1%)		
**Anorexia**				
Positive	31 (39.2%)	48 (60.8%)	**0.007**	− 0.215
Negative	48 (60.8%)	31 (39.2%)		
**Duration of symptoms**				
< 24 h	60 (75.9%)	23 (29.1%)	**< 0.001**	0.471
24–48 h	12 (15.2%)	40 (50.6%)		
> 48 h	7 (8.9%)	14 (20.3%)		
**Right lower quadrant pain with cough**				
Positive	36 (45.6%)	48 (60.8%)	0.560	− 0.152
Negative	43 (54.4%)	31 (39.2%)		
**Bowel sounds**				
Increased/metallic	1 (1.3%)	4 (5.1%)	**< 0.001**	0.537
Normal	39 (49.4%)	73 (94.2%)		
Absent	39 (49.4%)	2 (2.5%)		
**Defense**				
Absent	0 (0%)	24 (30.4%)	**< 0.001**	0.693
Mild	1 (1.3%)	10 (12.7%)		
Moderate	26 (32.9%)	41 (51.9%)		
Severe	52 (65.8%)	4 (5.1%)		
**Right lower quadrant sensitivity**				
Positive	79 (100%)	78 (98.7%)	1	0.08
Negative	0 (0%)	1 (1.3%)		
**Rebound**				
Positive	75 (94.8%)	58 (73.4%)	**< 0.001**	0.295
Negative	4 (5.1%)	21 (26.6%)		
**Rovsing’s sign**				
Positive	37 (46.8%)	16 (20.3%)	**0.001**	0.282
Negative	42 (53.2%)	63 (79.7%)		
**Pyrexia**				
Positive	16 (20.3%)	12 (15.2%)	0.532	0.066
Negative	63 (79.7%)	67 (84.8%)		
**Left shift in neutrophils**				
Positive	47 (59.5%)	23 (29.1%)	**< 0.001**	0.306
Negative	32 (40.5%)	56 (70.9%)		
**Negative urinalysis**				
Positive	79 (100%)	44 (55.7%)	**< 0.001**	0.533
Negative	0 (0%)	35 (44.3%)		
**Follow-up time**				
1 day	69 (87.3%)	53 (67.1%)	**0.003**	0.247
2 days	9 (11.4%)	25 (31.6%)		
3 days	1 (1.3%)	1 (1.3%)		

The data are shown in number and percentage format

*Phi or Cramer *V* coefficient is given as the effect size measure

**Fig. 1:**
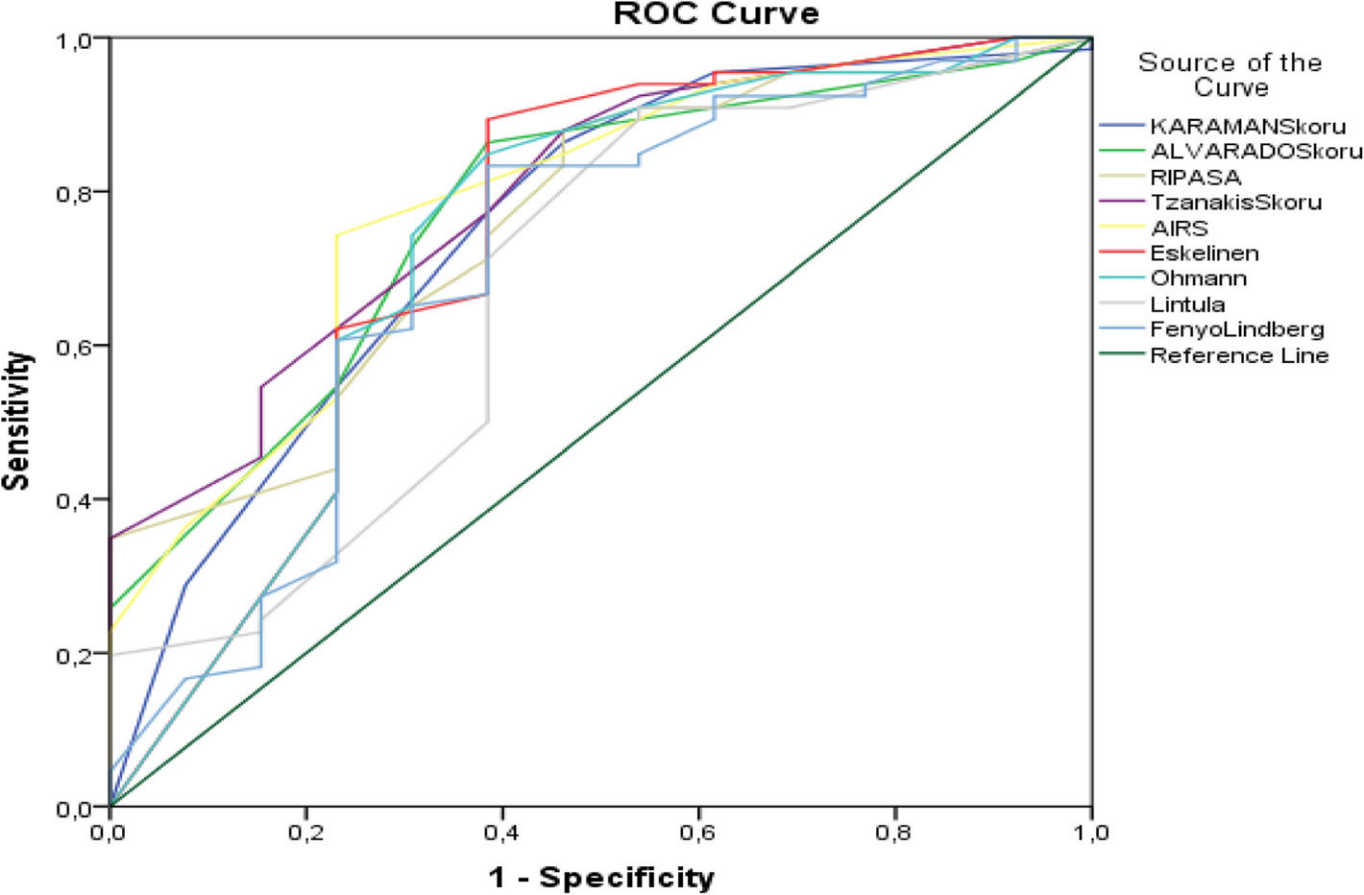
ROC curves for diagnostic performance of appendicitis scoring systems for non-pregnant women

We compared the efficacy of scoring systems for both groups. The Tzanakis system was the most efficacious among the scoring systems used in non-pregnant women. The positive predictive value (PPV) of the Tzanakis system was 90.6%, whereas the negative predictive value (NPV) was 46.7%. The sensitivity associated with the Tzanakis score was 87.8% and the specificity was 53.8%. Based on the area under the curve (AUC) analysis of predictive power, the Tzanakis score had the highest power in the non-pregnant group, followed by the AIR and Alvarado scores (Table 3) (Fig. 1).

**Table 3:** Distribution of appendicitis diagnostic performance criteria of scoring systems used in non-pregnant women

Variable(s)	AUC	***p***	PPV	NPV	Sensitivity	Specificity	Cutoff
**Karaman**	0.752	**0.004**	90.90	43.70	86.36	53.85	0.00
**Alvarado**	0.772	**0.002**	91.90	47.10	86.36	61.54	4.00
**RIPASA**	0.757	**0.004**	90.60	46.70	87.80	53.85	6.00
**Tzanakis**	0.794	**0.001**	90.60	46.70	87.80	53.85	6.00
**AIR**	0.787	**0.001**	94.20	37.00	74.24	76.92	4.00
**Eskelinen**	0.735	**0.008**	92.20	53.30	89.39	61.54	56.73
**Ohmann**	0.734	**0.008**	91.80	44.40	84.85	61.54	10.50
**Lintula**	0.675	**0.047**	89.60	50.00	90.91	46.15	8.00
**Fenyo-Lindberg**	0.705	**0.020**	91.70	42.10	83.33	61.54	− 33.00

**Fig. 2:**
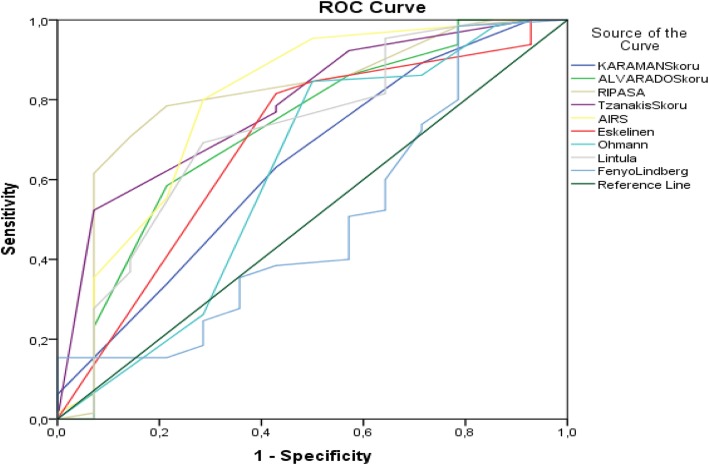
ROC curves for diagnostic performance of appendicitis scoring systems in pregnant women

RIPASA performed the best among the scoring systems used in pregnant patients. The PPV of this scoring method was 94.40%, its NPV was 44%, and its sensitivity and specificity were 78.46% and 78.57%, respectively. The AIR and Tzanakis systems were the second- and third-most efficacious, respectively. The PPV of the AIR score was 92.9%, its sensitivity was 80%, and its specificity was 71.4%. The PPV of the Tzanakis score was 97.1%, its sensitivity was 52.3%, and its specificity was 92.8% (Table 4) (Fig. 2).

**Table 4:** Distribution of appendicitis diagnostic performance criteria of scoring systems used in pregnant women

Variable(s)	AUC	***p***	PPV	NPV	Sensitivity	Specificity	Cutoff
**Karaman**	0.638	0.106	87.20	25.00	63.08	57.14	3.00
**Alvarado**	0.724	**0.009**	92.70	28.90	58.46	78.57	6.00
**RIPASA**	0.806	**0.000**	94.40	44.00	78.46	78.57	8.50
**Tzanakis**	0.786	**0.001**	97.10	29.50	52.31	92.86	13.00
**AIR**	0.795	**0.001**	92.90	43.50	80.00	71.43	6.00
**Eskelinen**	0.688	**0.028**	89.80	40.00	81.54	57.14	65.47
**Ohmann**	0.613	0.186	88.70	41.20	84.62	50.00	12.50
**Lintula**	0.723	**0.009**	91.80	33.30	69.23	71.43	19.00
**Fenyo-Lindberg**	0.498	0.980	25.00	14.70	1.54	78.57	− 36.00

## Discussion

Appendicitis is generally diagnosed based on clinical and laboratory findings, including the results of imaging analysis. However, the presence of numerous gynecological pathologies in female patients makes it challenging to diagnose acute appendicitis, particularly in pregnant patients [26]. For example, symptoms such as nausea and vomiting are common during both pregnancy and appendicitis, and laboratory findings also tend to be similar.

Radiological examination has high diagnostic value for acute appendicitis [27]. However, the main disadvantages of computed tomography are its teratogenic effect and high cost [28, 29]. The presence of positive abdominal ultrasonography (USG) findings in pregnant women with suspected appendicitis is sufficient to confirm the condition. In cases where appendicitis cannot be diagnosed using USG, magnetic resonance imaging provides high diagnostic accuracy in pregnant patients [30–32]. When using new scoring systems that combine clinical and imaging features, 95% of patients with uncomplicated appendicitis can be diagnosed correctly [33].

Delays in diagnosis and treatment of appendicitis may result in more complicated illness and increased rates of preterm labor, perinatal morbidity, mortality, and fetal loss [6–10]. The use of scoring systems helps to support imaging methods [34, 35]. CSS may be used in acute appendicitis to facilitate early diagnosis of the disease and prevent morbidity, and the Alvarado scoring system is one of the most commonly used systems for this situation [27]. Although the Alvarado system may be used in pregnant patients, its use has been extensively validated mainly in non-pregnant patients [36].

CSS aim to diagnose appendicitis by assessing signs, symptoms, and laboratory results. Systems use different variables. For example, the Tzanakis system includes USG results as a factor, the Lintula and Fenyo-Lindberg systems include gender, and the RIPASA and Ohmann systems include urinary symptoms. In our study, we assessed nine different scoring systems that are commonly used worldwide to diagnose appendicitis and compared their performance in pregnant and non-pregnant patients.

The presence of nausea, vomiting, and physiological leukocytosis during pregnancy makes it challenging to diagnose appendicitis, as does the fact that the position of the appendix changes during the gestation period [26]. In addition to our traditional knowledge, while rare publications are stating that the position of the appendix does not vary during pregnancy, publications with high volume are inevitably required to clarify this circumstance [37, 38]. This may explain why the negative appendectomy rate is approximately 35% in pregnant patients [39]. Currently, there is no scoring system that specifically evaluates appendicitis during pregnancy. Therefore, we evaluated the efficacy of existing scoring systems in pregnant patients. Based on AUC analysis, the scoring systems with the highest predictive power for non-pregnant women were the Tzanakis, AIR, and Alvarado systems, in that order. The Lintula and Fenyo-Lindberg scoring systems produced the lowest AUC values among the nine scoring systems. These results suggest that including USG findings (e.g., as in the Tzanakis system) is valuable for the diagnosis of appendicitis in non-pregnant patients.

For pregnant women, the RIPASA score had the highest predictive value, followed by the AIR and Tzanakis scores. Scoring systems that were heavily based on signs and detailed laboratory findings had greater predictive power in the pregnant group. Although we determined that the RIPASA system performed best in pregnant patients, its sensitivity and specificity were below 80%. Based on our findings, we intend to conduct further CSS research focused on the gestation period.

The potential limitations of our study included its retrospective design, the small number of cases, and the retrospective control group, which may not have been representative of the main group.

In summary, systems that include variables such as changes in the neutrophil count, negative urinary findings, Rovsing’s sign, rebound, severe abdominal defense, absence of bowel sounds, short symptom duration, and severe abdominal pain perform well in pregnant patients (Table 2). Systems that score gender and pain displacement are less efficacious for pregnant patients. Notably, with the progression of pregnancy, the gravid uterus may affect pain migration.

## Conclusion

Among the CSS evaluated, the RIPASA system was found to be the most suitable for pregnant patients, and this system may also help guide the use of imaging methods for pregnant patients in the clinic. Scoring systems tailored for acute appendicitis during the gestation period will improve treatment outcomes for both the mother and fetus.

## Data Availability

There is no additional data available to share with the readers. The datasets used and/or analyzed during the current study are available from the corresponding author on reasonable request.

## Abbreviations

ROC: Receiver operating characteristics
PPV: Positive predictive value
NPV: Negative predictive value
WBC: White blood count
VAS: Visual analog scale
AUC: Area under the curve
CT: Computed tomography
USG: Abdominal ultrasonography
MRI: Magnetic resonance imaging
CSS: Clinical scoring systems
NOM: Non-operative management
